# Exercise–Linked Irisin: Consequences on Mental and Cardiovascular Health in Type 2 Diabetes

**DOI:** 10.3390/ijms22042199

**Published:** 2021-02-23

**Authors:** Ricardo Augusto Leoni De Sousa, Alex Cleber Improta-Caria, Bruno Solano de Freitas Souza

**Affiliations:** 1 Multicenter Post Graduation Program in Physiological Sciences, Brazilian Society of Physiology, Federal University of the Valleys of Jequitinhonha and Mucuri, Diamantina, Minas Gerais CEP 39.000-000, Brazil; ricardoaugustoleonidesousa@gmail.com; 2 Neuroscience and Exercise Study Group (Grupo de Estudos em Neurociências e Exercício–GENE), Federal University of the Valleys of Jequitinhonha and Mucuri, Diamantina, Minas Gerais CEP 39.000-000, Brazil; 3 Post-Graduate Program in Medicine and Health, Faculty of Medicine, Federal University of Bahia, Bahia CEP 40.110-100, Brazil; aleximprotacaria@gmail.com; 4 Department of Physical Education in Cardiology, Brazilian Society of Cardiology, Bahia CEP 41.170-130, Brazil; 5 Center for Biotechnology and Cell Therapy, São Rafael Hospital, Bahia CEP 41.253-190, Brazil; 6 D’Or Institute for Research and Education (IDOR), Rio de Janeiro CEP 22.281-100, Brazil; 7 Gonçalo Moniz Institute, Oswaldo Cruz Foundation (FIOCRUZ), Bahia CEP 40.296-710, Brazil

**Keywords:** physical activity, insulin resistance, inflammation, cognitive decline, memory, dementia

## Abstract

Type 2 diabetes mellitus (T2DM) is a metabolic disorder associated with insulin resistance and hyperglycemia. Chronic exposure to a T2DM microenvironment with hyperglycemia, hyperinsulinemia, oxidative stress and increased levels of proinflammatory mediators, has negative consequences to the cardiovascular system and mental health. Therefore, atherosclerotic cardiovascular diseases (CVD) and mental health issues have been strongly associated with T2DM. Lifestyle modifications, including physical exercise training, are necessary to prevent T2DM development and its associated complications. It is widely known that the regular practice of exercise provides several physiological benefits to subjects with T2DM, such as managing glycemic and blood pressure levels. Different types of exercise, from aerobic to resistance training, are effective to improve mental health and cognitive function in T2DM. Irisin is a myokine produced in response to exercise, which has been pointed as a relevant mechanism of action to explain the benefits of exercise on cardiovascular and mental health in T2DM patients. Here, we review emerging clinical and experimental evidence about exercise-linked irisin consequences to cardiovascular and mental health in T2DM.

## 1. Introduction

Type 2 diabetes mellitus (T2DM) is a metabolic disorder characterized by the presence of insulin resistance that leads to hyperglycemia and increased hemoglobin A1c (HbA1c) levels [1,2]. The incidence of T2DM is increasing at alarming rates. Eighty million adults have prediabetes in the United States, and approximately 12% of the adults have diabetes, 90% to 95% of whom have T2DM [1]. The T2DM epidemic is one of the main causes of mortality and morbidity worldwide, where tissues such as muscle, fat and liver are less responsive or resistant to insulin [3]. The International Diabetes Federation estimates that almost 200 million people are undiagnosed worldwide and are, therefore, with a higher risk of developing several physiological complications [4]. Many of these people are pre-diabetic individuals. The term pre-diabetes is normally used to describe individuals who present impaired fasting glucose, and/or elevated glycosylated hemoglobin, and/or impaired glucose tolerance that did not reach or passed the T2DM parameters [5]. Pre-diabetes is a predictive of T2DM being associated with an increased risk for developing cardiovascular diseases (CVD) [6].

Atherosclerotic CVD and mental health issues have also been strongly associated to the development of T2DM [7,8]. Aging is considered to be one of the main risk factors for T2DM [9]. It has been reported that a sedentary behavior, commonly seen in subjects with T2DM, is associated with many deleterious health outcomes [10]. Preventing T2DM development and treating its associated consequences should focus on lifestyle modifications [4,8]. Among these lifestyle modifications, exercise is a proven form of preventing and managing T2DM [11].

It is widely known that the regular practice of exercise provides physiological benefits to subjects with T2DM, such as a better blood glucose and blood pressure control [12,13,14]. Different types of exercise, from aerobic to resistance training, have been shown to be effective to improve mental health and cognitive function in T2DM [15,16]. The isolated effect of exercise as prevention and treatment against T2DM is largely documented [13,14,15,16], however little is known about how irisin, a myokine produced in response to exercise, [17] acts on cardiovascular and mental health in T2DM.

Irisin is upregulated by aerobic and resistance exercise [18]. Exercise induces the expression of the nuclear transcriptional co-activator peroxisome proliferator-activated receptor-γ co- activator-1-α (PGC-1α) in skeletal muscle fibers [8]. In response to exercise, PGC-1α leads to the production of the fibronectin type III domain containing 5 (FNDC5) protein, which is cleaved and generates irisin. It has been reported that irisin modulates peripheral metabolism and increases the level of several anti-inflammatory proteins in the brain [19]. It is expected that exposure to hyperglycemia, hyperinsulinemia, oxidative stress and proinflammatory mediators, hallmark features of T2DM, might have consequences to cardiovascular and mental health. Here, we review emerging clinical and experimental evidence about exercise-linked irisin consequences to cardiovascular and mental health in T2DM.

## 2. Molecular Mechanisms of T2DM

**Figure 1:**
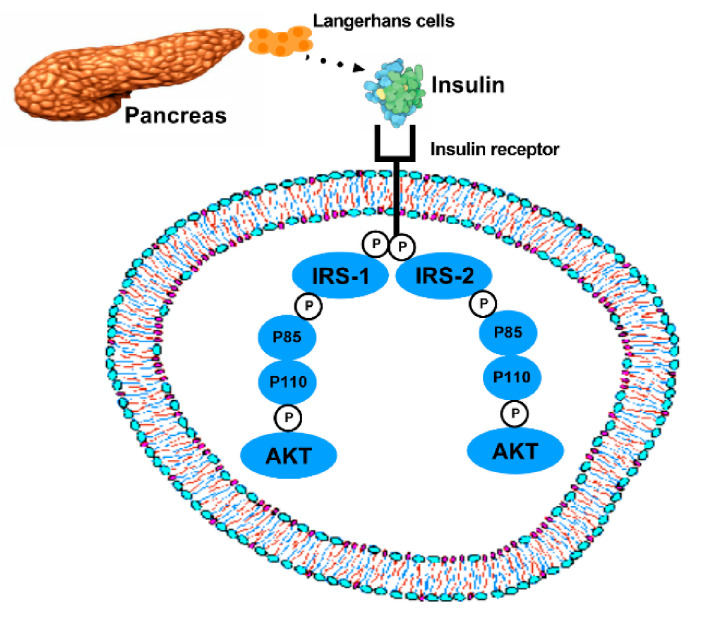
**PI3K pathway**. Insulin is produced in the Langerhans cells of the pancreas. Insulin binds to its receptor, which phosphorylates (p symbols) several substrates, but just two of them acts directly in glucose metabolism (IRS-1 and IRS-2). Thereafter, PI3K molecule, which is a dimer compound by P85 and P110 proteins, is also phosphorylated and activates AKT.

T2DM main feature is insulin resistance that occurs through phosphoidilinositol-3-kinase (PI3K) pathway impairment [2]. The PI3K pathway begins with a binding between the insulin hormone and its receptor. The insulin receptor has four subunits (2α and 2β). The α subunit inhibits the tyrosine kinase activity of the β subunit. Upon insulin binding to its receptor, the α subunit is inhibited and the β unit is allowed to exert its kinase activity. Insulin receptor substrates (IRS) are activated, but only IRS-1 and IRS-2 regulate glucose metabolism. IRS will activate PI3K, a dimmer compound by p85 and p110 proteins. Then, IRS phosphorylates p85, leading to the activation of p110 and PI3K phosphorylates activated kinase tyrosine (AKT) (Figure 1).

PI3K pathway activity is enough for allowing glucose transporter translocation [20]. The AKT phosphorylation leads glucose transporters to the cell membrane, uptaking glucose to the cells and playing an important role in peripheral tissues and brain in T2DM [2,8]. Glucose transporters are known mainly as GLUTs that ranges from 1–14, such as GLUT2 that allows easier diffusion of glucose in the intestine being also expressed in a subset of glutamatergic neurons in the hypothalamus [21], and GLUT4 that is the most important found in adipose and muscle tissues, the largest body tissues to respond to insulin [8]. It is well known that the regular practice of physical exercise increases glucose transporter protein GLUT4 in skeletal muscle of animal models with insulin resistance [22,23], and humans [2,8]. GLUT4 is the only one of its family that is not located in the cell membrane remaining in the vesicles into the cells, and it is necessary to activate PI3K pathway in order to facilitate AKT signaling to translocate the vesicle until the membrane to capture glucose [23,24], and its disturbance can contribute to the development of T2DM. Conversely, the intake of glucose into the brain is mediated mainly by GLUT1, which is expressed in endothelial cells ensuring delivery of glucose to glia cells, ependymal cells, and the choroid plexus, while GLUT3 is mainly concentrated in axons and dendrites [21].

The failure in PI3K pathway activation associated with insulin resistance may occur through different points of the pathway, as the binding of insulin to its receptor or along the signaling cascade [8]. The hallmark of insulin resistance in PI3K pathway is the inhibition of the insulin receptors and/or its substrates, especially the insulin receptor substrate 2 (IRS-2) and IRS-1, leading to hyperinsulinemia and hyperglycemia in the peripheral tissues, such as in muscle and adipose tissues [2,25]. A growing body of evidence have showed that insulin resistance can be also present in the brain and lead to the development of different conditions and pathologies, such as cognitive decline and memory loss [26,27,28,29].

The development of T2DM can upregulate inflammation markers and contribute to increase the production of reactive oxygen species (ROS) [30], which can react with lipids, proteins, nucleic acids, and several other molecules [31]. The effectiveness of physical exercise in diminishing ROS depends on the protocol adopted to the patients with T2DM [32]. Increased oxidative stress and inflammation will contribute to the development of insulin resistance and impaired insulin secretion [30]. Many different signaling pathways can be changed by the production of oxidative stress; by the formation of advanced glycation end products (AGEs); and by the secretion of pro-inflammatory cytokines [33,34]. Among the molecular mechanisms present in the pathomechanism of T2DM is important and valuable to evaluate the role of adipocytokines.

### The Role of Adipocytokines in T2DM

Adipose tissue dysfunction induces CVD through mechanisms that can be enhanced by T2DM [35]. The adipose tissue secretes many bioactive peptides/proteins, immune molecules and inflammatory mediators known as adipocytokines, which play important roles in the maintenance of energy homeostasis, appetite, glucose and lipid metabolism, insulin sensitivity, angiogenesis, immunity and inflammation [36]. Adipocytokines are involved in the pathogenesis of T2DM [35,37]. The adipocytokines are associated with the imbalance of glucose homeostasis include tumor necrosis factor-alpha (TNF-α), a number of different interleukins, monocyte chemoattractant protein-1, adiponectin, and leptin, among others [37].

There are newly discovered adipocytokines that contribute to the development of many different pathophysiological mechanisms, such as: (a) the enhancement of asprosin in the brain increases appetite and favors weight gain; (b) the elevation of asprosin levels in the pancreas decreases inuslin secretion and β cell survival; (c) the enhancement of lipocalin-2, omentin-1, and asprosin in the liver influences glucose release, hepatic inflammation, and lipid metabolism; (d) the elevation of the levels of lipocalin-2 and omentin-1 in the vessels also influences atherosclerosis, inflammation, and vascular remodeling [38]. Adipocytokines imbalance also contributes to the development of the pathogenesis and clinical outcome of mental disorders [39,40]. All negative metabolic changes made by adipocytokines imbalance can be prevented and fought by the regular practice of physical exercise [35,37,41].

A study conducted by Jorge et al. investigated the effects of 3 different modalities of exercise (aerobic, resistance and combined exercise) on metabolic control, insulin resistance, inflammatory markers, adipocytokines, and tissue expression of IRS–1 after 12 weeks of training among patients with T2DM [42]. Forty-eight patients with T2DM participated of this study and adiponectin, visfatin, and resistin, different types of adipocytokines, among other pro-inflammatory markers, were evaluated. All groups that underwent physical exercise protocols presented a decrease in lipid profile. Concomitantly, physical exercise also reduced blood pressure, fasting plasma glucose, postprandial plasma glucose, and C-reactive protein. These results suggest that physical exercise can reduce adipocytokines and several other pro-inflammatory biomarkers in patients with T2DM. Physical exercise fights the negative outcomes of adipocytokines imbalance mainly through the production and secretion of a myokine named Irisin [41,43].

## 3. Irisin

**Figure 2:**
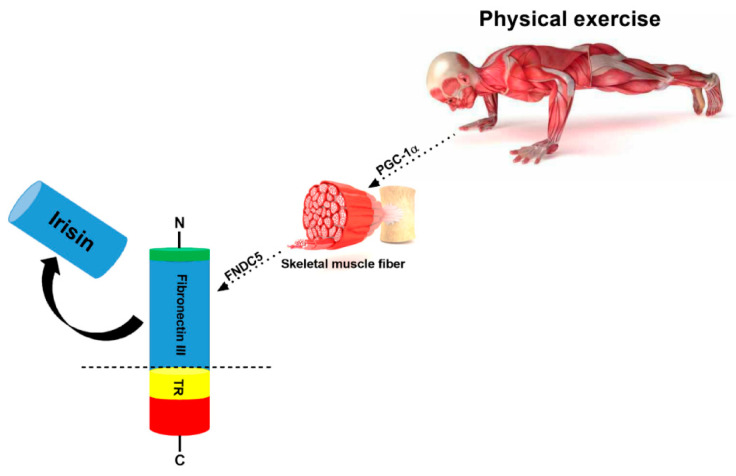
**Irisin is produced by exercise.** In response to exercise peroxisome proliferator, PGC-1α, leads to the production of FNDC5, a protein which contains an N-terminal signal sequence (green); fibronectin III (blue); a transmembrane region (TR) (yellow); a cytosolic region with a C-terminal tail (red). The proteolytic cleavage of mature FNDC5 results in the release of irisin, which corresponds to the fibronectin III domain with the signal sequence being removed.

Named after the ancient Greek goddess Iris, who was the messenger that delivered bad news from the gods [44], Irisin is an exercise-induced myokine that is released into the bloodstream as the result of the cleavage from the extracellular ectodomain of FNDC5 [45]. Schumacher et al. revealed the irisin structure and its biochemical characteristics, showing that this myokine is a dimer composed by an N-terminal fibronectin type-III-like region attached to a small C-terminal tail (Figure 2) [44,46].

Irisin is known for regulating thermogenesis and biogenesis of the brown adipose tissue [47]. In vitro experiments showed that higher levels of circulating irisin improve glucose tolerance and reduce insulin resistance [48]. In vivo experiments also showed that irisin is elevated after an exercise protocol and reverses cognitive decline [19]. Irisin levels are also negatively correlated with body mass index (BMI), waist circumference, and triglycerides in humans [41]. It has been reported that irisin could be therapeutic for CVD in T2DM [48]. Conversely, elevated plasma irisin has been reported to exist in T2DM being associated with indices of adiposity [43]. Due to these controversial information, understanding the molecular basis for exercise-linked irisin phenomena is, therefore, of considerable interest in T2DM.

## 4. Exercise-Linked Irisin: Consequences on Cardiovascular Health in T2DM

Circulating irisin is usually decreased in individuals with T2DM [49]. Zhang et al., showed that exercise induces positive changes in T2DM, including increased irisin levels [50]. The authors showed that exercise can beneficially impact the cardiovascular system leading to the elevation of uncoupling protein-1 (UCP-1) expression in white adipose tissue cells inducing conversion of these cells into brown type fat cells. The authors also revealed that production of irisin leads to phosphorylation of p38 mitogen-activated protein kinase (p38 MAPK) and activation of extracellular signal–related kinase (ERK), promoting an increase in expression of betatrophin, which generates proliferation and regeneration of beta-pancreatic cells. Increased serum levels of irisin, betatrophin and insulin were also seen in diabetic rats submitted to high-intensity interval training, which attenuated insulin resistance [51].

Exercise has been shown to improve insulin resistance [52], which is relevant since insulin resistance is an important pathophysiological process in T2DM associated with hyperinsulinemia, oxidation of fatty acids, and production of ROS, glucotoxicity and lipotoxicity [53]. T2DM induces activation of several cytokines, such as TNF-α, interleukin-6 (IL-6), interleukin 1-beta (IL-1β), growth factor-beta (TGF-β), which together lead to vascular endothelium and myocardial inflammation, cardiomyocyte hypertrophy, change in cell metabolism, cardiomyocyte death, fibroblasts activation and fibrosis, generating diabetic cardiomyopathy [54].

Exercise inhibits metabolic disorders and oxidative stress [55], attenuates endoplasmic reticulum stress [56] minimizes cardiac remodeling and improves the damage caused by ROS in the myocardium in diabetic cardiomyopathy [57], increases the expression of nitric oxide, improves microvessel diastolic function, promoting benefits for vascular endothelial cell function [58].

**Figure 3:**
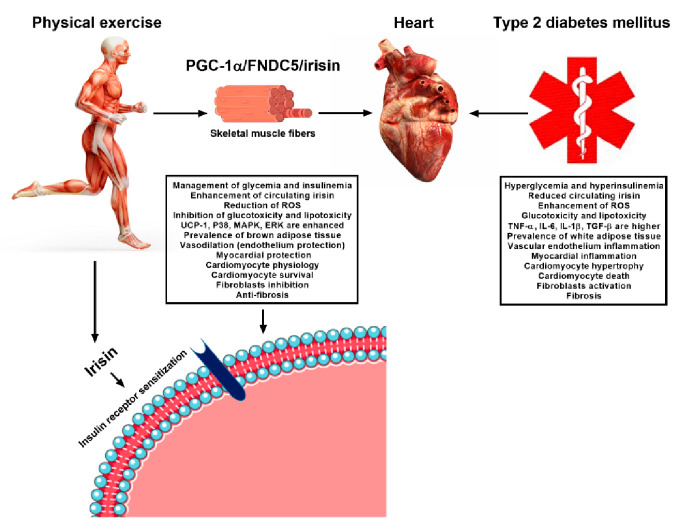
**Exercise-linked irisin: consequences on cardiovascular system in T2DM.** In T2DM there are many physiological changes that interfere with the cardiovascular system functioning like: hyperglycemia and hyperinsulinemia; reduced circulating irisin; enhancement of ROS; glucotoxicity and lipotoxicity; higher levels of pro-inflammatory proteins such as TNF-α, IL-6, IL-1b, TGF-β; prevalence of white adipose tissue; vascular endothelium inflammation; myocardial inflammation; cardiomyocyte hypertrophy; cardiomyocyte death; fibroblasts activation; fibrosis; and reduced insulin receptor sensitization. Although, the regular practice of exercise can inhibit these outcomes through many different ways like enhancing the UCP-1 expression in white adipose tissue cells inducing conversion of these cells into brown type fat cells. The production of irisin also leads to phosphorylation of p38 MAPK and activation of ERK, producing an increase in the expression of betatrophin, which generates proliferation and regeneration of beta-pancreatic cells.

Exercise reduces the expression of resistin and several proinflammatory cytokines in individuals with T2DM [59]. Furthermore, exercise promotes the reduction of cardiac fibrosis, improving myocardial function in diabetic cardiomyopathy [60]. All these beneficial mechanisms induced by exercise are associated with modifications in different molecules, including circulating microRNAs, which are post-transcriptional regulators of gene expression [52,61]. MicroRNAs can modulate different signaling pathways through exercise, and, therefore, contribute to the inhibition of insulin resistance in T2DM producing cardiovascular benefits in T2DM [61]. Finally, the improvement in insulin resistance through exercise is associated with increased expression of irisin, because it improves insulin receptor sensitization in the heart and skeletal muscle, favoring glucose uptake (Figure 3) [62].

## 5. Exercise-Linked Irisin: Consequences on Mental Health in T2DM

The effects of physical exercise training on insulin resistance are also associated with the prevention of cognitive decline and memory loss in both humans [63] and animal models [64]. Exercise also is capable to inhibit changes in mood and behavior in humans [65] and animal models [66]. In this context, animal models have been very useful for the investigation of molecular mechanisms, acute and long-term effects of exercise on mental health in T2DM. Several different animal models submitted to exercise reveal beneficial physiological effects, such as higher expression of PGC-1α/FNDC5/irisin that is associated to the improvement of inflammation and the increasing of mitochondrial membranes proteins [67].

### 5.1. Exercise-Linked Irisin: Consequences on Cognitive Function and Memory in T2DM

A recent study analyzed the behavior of human cells when exposed to irisin [19]. In the in vitro experiment, the authors described several important proteins to preserve and improve cognitive function and memory were higher in human cortical slices treated with irisin when compared to the Vehicle group, such as cyclic adenosine monophosphate (cAMP), protein kinase A (PKA), and cAMP-element binding protein (CREB). It has been reported that exercise can induce central expression of BDNF and other genes involved with neuroprotection and memory in mice [19,68]. Another recent study investigated whether irisin could protect neurons against Aβ oligomers, which are known for being the main cause of cognitive impairment and memory loss in Alzheimer’s disease [69]. The authors showed that irisin has neuroprotective effects on cultured neurons by regulating astrocytes.

Decline in cognitive function and memory loss have been reported among the consequences of T2DM to humans [6]. T2DM issues with memory and cognition are more prevalent among the elderly [70]. Espeland et al., evaluated non-demented men and women with T2DM, aged 70–89 years, who were sedentary and presented functional limitations [71]. The authors reported cognitive function benefits among participants with, but not without, diabetes. There are several studies that showed beneficial effects of exercise on cognitive function or memory in T2DM patients, but most of them did not analyze irisin levels in this population [72,73,74].

Lin et al., evaluated 133 Chinese patients, aged 45–75 years, with T2DM, where 59 patients were diagnosed with mild cognitive impairment (MCI) and 74 patients were included as healthy-cognition controls [75]. The main findings of this study revealed that patients with T2DM who had MCI presented significant higher levels of irisin, HbA1c, and insulin. Intringuily, the authors showed that patients with T2DM presented a poor glycemic control and higher plasma levels of irisin, which were correlated with cognitive decline on these individuals. Unfortunately, the levels of physical activity were not measured among these patients. The absence of measurement of irisin levels on the cerebrum spinal fluid and brain areas involved with cognitive function and memory can be viewed as a strong limitation of their results.

Regarding animal studies, Wang et al., assessed whether irisin was able to improve memory and cognitive performance in a diabetic mouse model [76]. The authors used 8-week-old male C57BL/6 for this study. The animals were randomly assigned into 4 groups: control, control plus irisin (0.5 mg/kg/day), streptozotocin (STZ) (150 mg/kg), and STZ plus irisin (0.5 mg/kg/day). The authors induced a single dose of STZ intraperitoneally to establish the diabetic mouse model. Cortical and spatial memory were assessed through the novel object recognition task and the Y-maze, respectively. It was found upregulated levels of glial fibrillary acid protein (GFAP), a biomarker for astrocytes, reduced synaptic protein expression, and increased levels of IL-1β and IL-6. The irisin treatment also inhibited the activation of P38, STAT3, and NFκB (proteins responsive to stress stimuli) on the diabetic mice. Decline in cognitive function and memory was seen in the diabetic mice, but could be avoided by irisin cotreatment.

De Sousa et al. used a resistance training protocol in Wistar rats induced to T2DM by dexamethasone (0.5 mg/kg/day, i.p) [16]. Four weeks of high-intensity resistance training were enough to inhibit cognitive decline by stimulating the activation of IRS-1 and reducing the activation of glycogen synthase kinase 3 beta (GSKβ). A recent study evaluated the effects of exercise on the PGC-1α/FNDC5/irisin pathway using different models of Alzheimer’s disease, which is considered a type 3 diabetes due to the presence of insulin resistance in the brain, in mice [12]. Mice performed a swimming training for 4 weeks. The exercised mice presented lower cognitive and memory deficits associated to higher levels of irisin, FNDC5, PGC-1α, and BDNF in the brain. Interestingly, when the exercised animals received an anti-FNDC5 antibody the beneficial effects of exercise on cognition and memory were abolished. Intringuily, the plasma levels of FNDC5/irisin were unaltered. All these findings together reveal that plasma levels of irisin are not enough to predict cognitive decline and a more invasive technique is necessary to evaluate the irisin levels in the brain.

**Figure 4:**
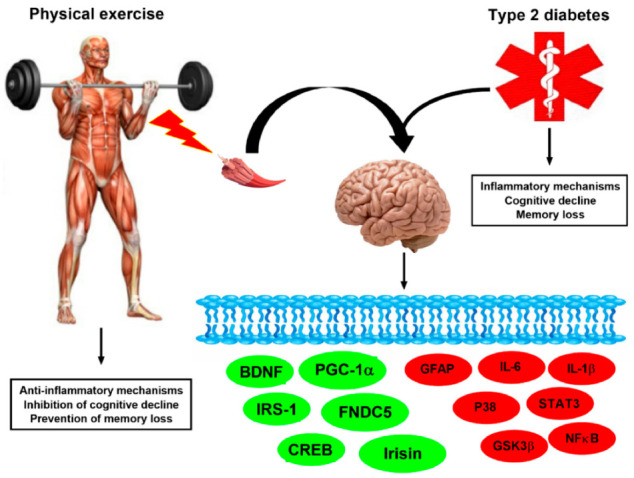
**Exercise-linked irisin: potential molecular mechanisms on cognitive function and memory in T2DM.** Proteins that are upregulated due to exercise in green (PGC-1α, FNDC5, irisin, BDNF, CREB, and IRS-1); proteins downregulated or that maintain their levels unaltered are in red (GFAP, IL-1β, IL-6, P38, STAT3, NFκB, and GSKβ) in T2DM. Thus, anti-inflammatory mechanisms occur due to exercise in T2DM. These potential exercise effects are suggested to protect against cognitive decline and memory loss in T2DM.

The molecular mechanisms by which exercise positively influences the cognitive function and memory in T2DM seems to involve upregulation of PGC-1α, FNDC5, irisin, BDNF, CREB, and IRS-1. Nevertheless, it seems to exist downregulation or maintenance of the normal levels of GFAP, IL-1β, IL-6, P38, STAT3, NFκB, and GSKβ (Figure 4).

### 5.2. Exercise-Linked irisin: Consequences on Depression and Anxiety in T2DM

Depression is the most common neuropsychiatric disorder and affects millions of people worldwide [77]. Han et al., 2019 investigated whether there could be a possible link between energy homeostasis management and coronary heart disease patients with a depressive-like behavior [78]. The authors found that the role of energy homeostasis in the susceptibility to depression in patients with coronary heart disease. However, the interaction between irisin and BDNF was capable to trigger the imbalance of energy homeostasis that occurs in depression on these patients. Irisin has been used for co-treatment with propofol in animal model (mice) to evaluate whether irisin could prevent the depressive-like behavior [79]. The authors showed that irisin significantly inhibited the increase of cytokines in astrocyte cultures exposed to propofol (in vitro). Irisin was also capable to reduce significantly the depressive-like behavior in mice. Unfortunately, there are not studies to date that have evaluated exercise-linked irisin effects on depression in diabetic subjects or animal models induced to T2DM. Nevertheless, there is evidence showing that exercise can prevent and treat the depressive-like behavior in diabetic subjects [80] and on animals models of T2DM [81].

Cassilhas et al., investigated whether a resistance training protocol could inhibit the development of an anxious profile and a depressive-like behavior in the elderly [65]. The authors revealed that the resistance training protocol was enough to inhibit anxiety and depression, and insulin growing factor 1 (IGF-1) at serum concentration in elderly individuals. Uysal et al., also showed, using animal model (mice), that regular aerobic exercise correlates with reduced anxiety and increased levels of irisin in brain [82]. Intringuily, we did not find any studies to date that have evaluated exercise-linked irisin effects on anxiety in diabetic subjects or animal models induced to T2DM either. However, there is evidence showing that being physically inactive is strongly associated with anxiety in diabetic subjects [83]. Animal models of T2DM who are physically inactive also shows the development of the anxious profile [84]. Possibly, there would be an important role of irisin on the treatment of mood disorders, such as depression and anxiety, that needs to be investigated in T2DM.

## 6. Exercise in T2DM: Types, Variables and Outcomes

It is widely known that physical activity and/or regular exercise routine are crucial for body and brain healthy [85,86]. Physical activity can be considered as any routine of body movement, such as gardening, that leads you to burn more calories than when you are into a rest condition. On the other hand, a regular exercise routine includes a structured methodology that aims to enhance muscular tone or endurance capacity [87]. Despite the different concepts regarding physical activity and the regular exercise routine, their outcomes frequently achieve a similar overall benefit [85,87,88]. There are many known effects of physical activity and the regular practice of exercise, such as reducing the risk of T2DM, obesity, Alzheimer’s disease, and other diseases and chronic conditions [85,88]. Nevertheless, the regular practice of exercise presents a higher level of complexity than physical activity.

Exercise routine can include different types of exercise, such as aerobic (endurance) and resistance (strength) training [87]. Exercise is prescribed based on different variables, such as volume, frequency, intensity, and duration. Endurance training is characterized by the execution of exercises with greater utilization of oxygen, predominant recruitment of red fibers, also known as type I fibers, or fibers of slow contraction [87,89]. Strength training is characterized by the execution of exercises against any external force, which might be the individual’s body mass, air resistance, or elastic resistance, with predominant recruitment of white fibers, also known as type I fibers, or fibers of rapid contraction [89].

According to the World Health Organization (WHO) recommendations, adults should perform strength training at a minimum weekly frequency of 2 days [90]. Endurance training should be performed for at least 150 min at moderate intensity per week, or for 75 min at high intensities, at least 5× weekly [90,91]. The standards of medical care in diabetes in 2020, published by the American Diabetes Association (ADA), adds that endurance activity could be complemented by strength training. Thus, endurance and strength training should be used in the prevention and treatment of T2DM [92].

Many individuals with T2DM are overweight or obese and losing at least 5% of their body mass is needed to achieve positive outcomes in glycemic control, lipids profile, and blood pressure management [93]. However, more intensive weight loss goals might be necessary (i.e., 15%) in order to improve the benefits depending on need, feasibility, and safety [94]. According to the actual standards of medical care in diabetes, individuals with T2DM should be engaged in exercise programs, follow a healthy eating plan, take their medications, and metabolic surgery should be also considered to achieve and maintain weight loss goals and lower CVD risk factors [92].

A recent study evaluated the effects of moderate-intensity at different workloads during the strength sessions in T2DM individuals [12]. The sample was composed by 40 male volunteers. There were initially two groups: nondiabetics (ND; control group) and hypertensive diabetics (D), who were later subdivided into 4 groups, consisting of 10 individuals each, separated according to the percentage of the maximum intensity session. The groups performed the RE protocols at 60% (ND60 and D60) or at 75% (ND75, and D75) of the 1 repetition maximum test (1RM). The authors adopted as inclusion criteria to be a male between 40 and 60 years old, and the participants could not be doing regular physical activity during the last six months. The main results in this study showed that the highest moderate intensity (75% 1RM) revealed a better glucose uptake from the diabetic group. In addition, independently of the strength exercise intensity, the moderate exercises sessions were well capable to reduce the blood pressure of the diabetic groups, presenting a notable tendency to a better reduction in the higher strength exercise intensity. The hormonal response after strength exercises was suitable for a good exercise recovery, mainly for exercise performed at 75% 1RM, which resulted in an elevated indirect marker of muscle damage. These results together indicate that acute strength exercise at 60% or 75% of 1RM can be used as a non-pharmacological tool to help the management of both pathologic conditions combined (T2DM and hypertension).

The study of Lee et al. [95] investigated the effects of 12 weeks regular endurance exercise on BDNF and inflammatory factors in juvenile obesity and T2DM. All individuals who participated of the study were between the ages of 13 and 19 years, and there were 17 boys and 9 girls. The exercise test was conducted using a treadmill exercise test. The protocol used involves steadily increasing the exercise level on the treadmill until the target heart rate (THR) reaches 85% of the maximum oxygen consumption (VO2_max_). There were no subjects that exhibited cardiac abnormalities or elevated blood pressure before reaching the THR. In order to prevent accidents, participants were asked to subjectively identify the intensity of exercise on the Borg scale of rate of perceived exertion [96]. The endurance exercise training was conducted for a total of 40–60 min per session, 3 sessions a week, for 12 weeks. Each session was preceded and followed by a 5-min warm up and cool down, respectively. The main findings in this study revealed that the juveniles with obesity and T2DM exhibited reduced levels of resting neurotrophic factors, such as BDNF and its receptor (TrkB) at baseline, but after the 12 weeks of endurance exercise, there was a significant increase in the BDNF levels. Thus, we suggest that acute or chronic programs of endurance or strength exercises can positively contribute to the improvement of the general health of T2DM individuals, and lower the risk of CVD or developing mental health issues.

There is another type of endurance training that is quite new if compared to the regular usage of endurance training, and has been very used to prevent or treat pathologies using human and animal models that is the high-intensity interval training (HIIT) [97,98]. HIIT consists in short sessions of intermittent endurance bouts, which is performed with an “all-out” effort and/or adding a lighter recovering period from each single bout [99,100]. HIIT volume is usually 90% lower than what is commonly used at endurance training, and also the time spent to perform the entire HIIT session is, often, 75% shorter [100]. Mendes et al. recently compared the acute effects of HIIT versus moderate-intensity continuous training (MICT) on glycemic control in middle-aged and older patients with T2DM [101]. Fifteen patients with T2DM, who were volunteers to participate of the study (60.25 ± 3.14 years; glycated hemoglobin 7.03 ± 0.33%; medicated with metformin and/or gliptins), were inserted into a randomized controlled crossover trial. The participants underwent 3 different experimental conditions (treadmill walking HIIT session (5 × (3 min at 70% of heart rate reserve (HRR) + 3 min at 30% HRR)); treadmill walking MICT session (30 min at 50% HRR); and a control session of rest (CON)) in random order and in the postprandial state. The authors found that treadmill walking HIIT seems a safe and more effective exercise strategy on immediate acute glycemic control compared with MICT in middle-aged and older patients with T2DM under therapy with metformin and/or gliptins. Nevertheless, we suggest that HIIT needs to be tested in different conditions involving T2DM individuals before starting a routine to this special group.

Physical inactivity is associated with deleterious health outcomes, such as the worsening of T2DM, increased blood pressure, and many others [10]. Despite of existing massive evidence that a single bout of endurance or strength exercise is capable to improve glycemic profiles in individuals with T2DM [102], we did not identify any studies in scientific literature that have evaluated acute effects of the both types of exercise on memory. Thus, there is a lack of research studies involving acute effects of endurance and strength training, and also the long-term effects of the strength exercise on memory of subjects with T2DM. The common sense about chronic endurance and strength training recommend both types of exercise to T2DM individuals [103]. Nevertheless, it is also important to remind when looking to exercise variables, such as the type of exercise (resistance or endurance), frequency (acute and chronic protocols), volume (low, moderate, high), duration (period of time of each session) and intensity (low, moderate, high) should influence and present different results [104,105,106]. Regarding the usage of animal models to evaluate different exercise-linked irisin benefits, we suggest that exercise protocols, independently of being endurance or strength training, must be adapted according to the specie, metabolism and life span of the animal [29].

## 7. Conclusions

Exercise-linked irisin is associated to a better cardiovascular health and improvement of the cognitive function and memory in T2DM subjects, and also in animal models induced to T2DM. The gap in scientific literature towards exercise-linked irisin to consequences on depression and anxiety in T2DM urges to be investigated. Exercise-linked irisin is a promisor target to investigate the molecular mechanisms about how exercise changes cardiovascular and brain function in T2DM and many other pathologies.

## References

[1] Arnett D.K., Blumenthal R.S., Albert M.A., Buroker A.B., Goldberger Z.D., Hahn E.J., Michos E.D., McEvoy J.W., Miedema M.D., Himmelfarb C.D. (2019). 2019 ACC / AHA Guideline on the Primary Prevention of Cardiovascular Disease. J. Am. Coll. Cardiol..

[2] De Sousa R.A.L., Harmer A.R., Freitas D.A., Mendonça V.A., Lacerda A.C.R., Leite H.R. (2020). An update on potential links between type 2 diabetes mellitus and Alzheimer’s disease. Mol. Biol. Rep..

[3] Saltiel A.R., Kahn C.R. (2001). Insulin signalling and the regulation of glucose and lipid metabolism. Nature.

[4] International Diabetes Federation (2017). Recommendations for Managing Type 2 Diabetes in Primary Care.

[5] Kelly A.S., Bergenstal R.M., Gonzalez-Campoy J.M., Katz H., Bank A.J. (2012). Effects of exenatide vs. metformin on endothelial function in obese patients with pre-diabetes: A randomized trial. Cardiovasc. Diabetol..

[6] Roriz-Filho J.S., Sá-Roriz T.M., Rosset I., Camozzato A.L., Santos A.C., Chaves M.L.F., Moriguti J.C., Roriz-Cruz M. (2009). (Pre)diabetes, brain aging, and cognition. Biochim. Biophys. Acta Mol. Basis Dis..

[7] Bordier L., Doucet J., Boudet J., Bauduceau B. (2014). Update on cognitive decline and dementia in elderly patients with diabetes. Diabetes Metab..

[8] Sousa R.A.L. (2018). de Brief report of the effects of the aerobic, resistance, and high-intensity interval training in type 2 diabetes mellitus individuals Diabetes mellitus. Int. J. Diabetes Dev. Ctries..

[9] Kitada M., Ogura Y., Monno I., Koya D. (2019). Sirtuins and Type 2 Diabetes: Role in Inflammation, Oxidative Stress, and Mitochondrial Function. Front. Endocrinol..

[10] Tremblay M.S., Colley R.C., Saunders T.J., Healy G.N., Owen N. (2010). Physiological and health implications of a sedentary lifestyle. Appl. Physiol. Nutr. Metab..

[11] Tompkins C.L., Soros A., Sothern M.S., Vargas A. (2009). Effects of Physical Activity on Diabetes Management and Lowering Risk For Type 2 Diabetes. Am. J. Health Educ..

[12] De Sousa R.A.L., Hagenbeck K.F., Arsa G., Pardono E. (2020). Moderate / high resistance exercise is better to reduce blood glucose and blood pressure in middle-aged diabetic subjects. Rev. Bras. Educ. Física Esporte.

[13] Ranasinghe C., Hills A.P., Constantine G.R., Finlayson G., Katulanda P., King N.A. (2018). Study protocol: A randomised controlled trial of supervised resistance training versus aerobic training in Sri Lankan adults with type 2 diabetes mellitus: SL-DART study. BMC Public Health.

[14] Zisser H., Sueyoshi M., Krigstein K., Szigiato A., Riddell M.C. (2012). Advances in exercise, physical activity and diabetes mellitus. Int. J. Clin. Pract..

[15] Feter N., Spanevello R.M., Soares M.S.P., Spohr L., Pedra N.S., Bona N.P., Freitas M.P., Gonzales N.G., Ito L.G.M.S., Stefanello F.M. (2019). How does physical activity and different models of exercise training affect oxidative parameters and memory?. Physiol. Behav..

[16] De Sousa R.A.L., Caria A.C.I., De Jesus Silva F.M., Magalhães C.O.D., Freitas D.A., Lacerda A.C.R., Mendonça V.A., Cassilhas R.C., Leite H.R. (2020). High-intensity resistance training induces changes in cognitive function, but not in locomotor activity or anxious behavior in rats induced to type 2 diabetes. Physiol. Behav..

[17] Benedini S., Dozio E., Invernizzi P.L., Vianello E., Banfi G., Terruzzi I., Luzi L., Romanelli M.M.C. (2017). Irisin: A Potential Link between Physical Exercise and Metabolism—An Observational Study in Differently Trained Subjects, from Elite Athletes to Sedentary People. J. Diabetes Res..

[18] Reza M.M., Subramaniyam N., Sim C.M., Ge X., Sathiakumar D., McFarlane C., Sharma M., Kambadur R. (2017). Irisin is a pro-myogenic factor that induces skeletal muscle hypertrophy and rescues denervation-induced atrophy. Nat. Commun..

[19] Lourenco M.V., Frozza R.L., de Freitas G.B., Zhang H., Kincheski G.C., Ribeiro F.C., Gonçalves R.A., Clarke J.R., Beckman D., Staniszevski A. (2019). Exercise-linked FNDC5/irisin rescues synaptic plasticity and memory defects in Alzheimer’s models. Nat. Med..

[20] Czech M.P., Corvera S. (1999). Signaling Mechanisms That Regulate Glucose Transport. J. Biol. Chem..

[21] Camandola S., Mattson M. (2017). Brain metabolism in health, aging, and neurodegeneration. EMBO J..

[22] Friedman J.E., Sherman W.M., Reed M.J., Elton C.W., Dohm G.L. (1990). Exercise training increases glucose transporter protein GLUT-4 in skeletal muscle of obese Zucker (fa/fa) rats. FEBS Lett..

[23] Hardin D.S., Dominguez H., Timothy W. (1993). Muscle Group-Specific Regulation of Glut 4 Glucose Transporters in Control, Diabetic, and Insulin Treated Diabetic Rats. Metabolism.

[24] Egawa T., Tsuda S., Ma X., Hamada T., Hayashi T. (2011). Caffeine modulates phosphorylation of insulin receptor substrate-1 and impairs insulin signal transduction in rat skeletal muscle. J. Appl. Physiol..

[25] Sousa R.A.L. (2018). de Gestational diabetes is associated to the development of brain insulin resistance in the offspring. Int. J. Diabetes Dev. Ctries..

[26] Yi S.S. (2015). Effects of exercise on brain functions in diabetic animal models. World J. Diabetes.

[27] Ferreira L.S.S., Fernandes C.S., Vieira M.N.N., De-Felice F.G. (2018). Insulin resistance in Alzheimer’s disease. Front. Neurosci..

[28] Folch J., Ettcheto M., Busquets O., Sánchez-López E., Castro-Torres R.D., Verdaguer E., Manzine P.R., Poor S.R., García M.L., Olloquequi J. (2018). The implication of the brain insulin receptor in late onset Alzheimer’s disease dementia. Pharmaceuticals.

[29] De Sousa R.A.L., Rodrigues C.M., Mendes B.F., Improta-caria A.C., Peixoto M.F.D., Cassilhas R.C. (2020). Physical exercise protocols in animal models of Alzheimer’ s disease: A systematic review. Metab. Brain Dis..

[30] Luc K., Schramm-Luc A., Guzik T.J., Mikolajczyk T.P. (2019). Oxidative stress and inflammatory markers in prediabetes and diabetes. J. Physiol. Pharmacol..

[31] Huang W., Zhang X., Chen W. (2016). Role of oxidative stress in Alzheimer’s disease (Review). Biomed. Rep..

[32] Poblete-Aro C., Russell-Guzmán J., Parra P., Soto-Muñoz M., Villegas-González B., Cofré-Bola-Dos C., Herrera-Valenzuela T. (2018). Exercise and oxidative stress in type 2 diabetes mellitus. Rev. Med. Chil..

[33] Volpe C.M.O., Villar-Delfino P.H., Dos Anjos P.M.F., Nogueira-Machado J.A. (2018). Cellular death, reactive oxygen species (ROS) and diabetic complications review-Article. Cell Death Dis..

[34] Yang G., Sau C., Lai W., Cichon J., Li W. (2018). Reactive Oxygen Species in Metabolic and Inflammatory Signaling. Circ. Res..

[35] Chait A., den Hartigh L.J. (2020). Adipose Tissue Distribution, Inflammation and Its Metabolic Consequences, Including Diabetes and Cardiovascular Disease. Front. Cardiovasc. Med..

[36] Maximus P.S., Al Achkar Z., Hamid P.F., Hasnain S.S., Peralta C.A. (2020). Adipocytokines: Are they the Theory of Everything?. Cytokine.

[37] Banerjee A., Singh J. (2020). Remodeling adipose tissue inflammasome for type 2 diabetes mellitus treatment: Current perspective and translational strategies. Bioeng. Transl. Med..

[38] Kim J.A., Choi K.M. (2020). Newly Discovered Adipokines: Pathophysiological Link Between Obesity and Cardiometabolic Disorders. Front. Physiol..

[39] Wędrychowicz A., Zajac A., Pilecki M., Koscielniak B., Tomasik P.J. (2014). Peptides from adipose tissue in mental disorders. World J. Psychiatry.

[40] Weber-Hamann B., Kratzsch J., Kopf D., Lederbogen F., Gilles M., Heuser I., Deuschle M. (2007). Resistin and adiponectin in major depression: The association with free cortisol and effects of antidepressant treatment. J. Psychiatr. Res..

[41] Elizondo-Montemayor L., Gonzalez-Gil A.M., Tamez-Rivera O., Toledo-Salinas C., Peschard-Franco M., Rodríguez-Gutiérrez N.A., Silva-Platas C., Garcia-Rivas G. (2019). Association between irisin, hs-CRP, and metabolic status in children and adolescents with type 2 diabetes mellitus. Mediat. Inflamm..

[42] Jorge M.L.M.P., De Oliveira V.N., Resende N.M., Paraiso L.F., Calixto A., Diniz A.L.D., Resende E.S., Ropelle E.R., Carvalheira J.B., Espindola F.S. (2011). The effects of aerobic, resistance, and combined exercise on metabolic control, inflammatory markers, adipocytokines, and muscle insulin signaling in patients with type 2 diabetes mellitus. Metabolism.

[43] Rana K.S., Pararasa C., Afzal I., Nagel D.A., Hill E.J., Bailey C.J., Griffiths H.R., Kyrou I., Randeva H.S., Bellary S. (2017). Plasma irisin is elevated in type 2 diabetes and is associated with increased E-selectin levels. Cardiovasc. Diabetol..

[44] Buscemi S., Corleo D., Buscemi C., Giordano C. (2018). Does iris(in) bring bad news or good news?. Eat. Weight Disord..

[45] Boström P., Wu J., Jedrychowski M.P., Korde A., Ye L., Lo J.C., Rasbach K.A., Boström E.A., Choi J.H., Long J.Z. (2012). A PGC1a dependent myokine that derives browning of white fat and thermogenesis. Nature.

[46] Schumacher M.A., Chinnam N., Ohashi T., Shah R.S., Erickson H.P. (2013). The structure of Irisin reveals a novel intersubunit β-sheet fibronectin type III (FNIII) dimer: Implications for receptor activation. J. Biol. Chem..

[47] Puigserver P., Wu Z., Park C.W., Graves R., Wright M., Spiegelman B.M. (1998). A Cold-Inducible Coactivator of Nuclear Receptors Linked to Adaptive Thermogenesis. Cell.

[48] Lu J., Xiang G., Liu M., Mei W., Xiang L., Dong J. (2015). Irisin protects against endothelial injury and ameliorates atherosclerosis in apolipoprotein E-Null diabetic mice. Atherosclerosis.

[49] Kurdiova T., Balaz M., Vician M., Maderova D., Vlcek M., Valkovic L., Srbecky M., Imrich R., Kyselovicova O., Belan V. (2014). Effects of obesity, diabetes and exercise on Fndc5 gene expression and irisin release in human skeletal muscle and adipose tissue: In vivo and in vitro studies. J. Physiol..

[50] Zhang Y., Li R., Meng Y., Li S., Donelan W., Zhao Y., Qi L., Zhang M., Wang X., Cui T. (2014). Irisin stimulates browning of white adipocytes through mitogen-activated protein kinase p38 MAP kinase and ERK MAP kinase signaling. Diabetes.

[51] Amri J., Parastesh M., Sadegh M., Latifi S.A., Alaee M. (2019). High-intensity interval training improved fasting blood glucose and lipid profiles in type 2 diabetic rats more than endurance training; Possible involvement of irisin and betatrophin. Physiol. Int..

[52] Liu S.X., Zheng F., Xie K.L., Xie M.R., Jiang L.J., Cai Y. (2019). Exercise Reduces Insulin Resistance in Type 2 Diabetes Mellitus via Mediating the lncRNA MALAT1/MicroRNA-382-3p/Resistin Axis. Mol. Ther. Nucleic Acids.

[53] Boudina S., Abel E.D. (2007). Diabetic cardiomyopathy revisited. Circulation.

[54] Frati G., Schirone L., Chimenti I., Yee D., Biondi-Zoccai G., Volpe M., Sciarretta S. (2017). An overview of the inflammatory signalling mechanisms in the myocardium underlying the development of diabetic cardiomyopathy. Cardiovasc. Res..

[55] Mahmoud R., Wainwright S.R., Galea L.A.M. (2016). Sex hormones and adult hippocampal neurogenesis: Regulation, implications, and potential mechanisms. Front. Neuroendocr..

[56] Chengji W., Xianjin F. (2019). Exercise protects against diabetic cardiomyopathy by the inhibition of the endoplasmic reticulum stress pathway in rats. J. Cell. Physiol..

[57] Gimenes C., Gimenes R., Rosa C.M., Xavier N.P., Campos D.H.S., Fernandes A.A.H., Cezar M.D.M., Guirado G.N., Cicogna A.C., Takamoto A.H.R. (2015). Low Intensity Physical Exercise Attenuates Cardiac Remodeling and Myocardial Oxidative Stress and Dysfunction in Diabetic Rats. J. Diabetes Res..

[58] Cohen N.D., Dunstan D.W., Robinson C., Vulikh E., Zimmet P.Z., Shaw J.E. (2008). Improved endothelial function following a 14-month resistance exercise training program in adults with type 2 diabetes. Diabetes Res. Clin. Pract..

[59] Kadoglou N.P., Perrea D., Iliadis F., Angelopoulou N., Liapis C., Alevizos M. (2007). Exercise reduces resistin and inflammatory cytokines in patients with type 2 diabetes. Diabetes Care.

[60] Wang H., Bei Y., Lu Y., Sun W., Liu Q., Wang Y., Cao Y., Chen P., Xiao J., Kong X. (2015). Exercise prevents cardiac injury and improves mitochondrial biogenesis in advanced diabetic cardiomyopathy with PGC-1α and Akt activation. Cell. Physiol. Biochem..

[61] Caria A.C.I., Nonaka C.K.V., Pereira C.S., Soares M.B.P., Macambira S.G., de Souza B.S.F. (2018). Exercise Training-Induced Changes in MicroRNAs: Beneficial Regulatory Effects in Hypertension, Type 2 Diabetes, and Obesity. Int. J. Mol. Sci..

[62] Gizaw M., Anandakumar P., Debela T. (2017). A Review on the Role of Irisin in Insulin Resistance and Type 2 Diabetes Mellitus. J. Pharmacopunct..

[63] Baker L.D., Frank L.L., Foster-Schubert K., Green P.S., Wilkinson C.W., McTiernan A., Plymate S.R., Fishel M.A., Watson G.S., Cholerton B.A. (2010). Effects of aerobic exercise on mild cognitive impairment: A controlled trial. Arch. Neurol..

[64] Shima T., Matsui T., Jesmin S., Okamoto M., Soya M., Inoue K., Liu Y.F., Torres-Aleman I., McEwen B.S., Soya H. (2017). Moderate exercise ameliorates dysregulated hippocampal glycometabolism and memory function in a rat model of type 2 diabetes. Diabetologia.

[65] Cassilhas R.C., Antunes H.K.M., Tufik S., de Mello M.T. (2010). Mood, Anxiety, and Serum IGF-1 in Elderly Men Given 24 Weeks of High Resistance Exercise. Percept. Mot. Skills.

[66] Park H., Lee J., Cho H., Park S., Kim T. (2017). Physical exercise ameliorates mood disorder-like behavior on high fat diet- induced obesity in mice. Psychiatry Res..

[67] Botta A., Laher I., Beam J., DeCoffe D., Brown K., Halder S., Devlin A., Gibson D.L., Ghosh S. (2013). Short Term Exercise Induces PGC-1α, Ameliorates Inflammation and Increases Mitochondrial Membrane Proteins but Fails to Increase Respiratory Enzymes in Aging Diabetic Hearts. PLoS ONE.

[68] Wrann C.D., White J.P., Salogiannnis J., Laznik-Bogoslavski D., Wu J., Ma D., Lin J.D., Greenberg M.E., Spiegelman B.M. (2013). Exercise induces hippocampal BDNF through a PGC-1α/FNDC5 pathway. Cell Metab..

[69] Wang K., Li H., Wang H., Wang J.H., Song F., Sun Y. (2018). Irisin exerts neuroprotective effects on cultured neurons by regulating astrocytes. Mediat. Inflamm..

[70] Bourdel-Marchasson I., Lapre E., Laksir H., Puget E. (2010). Insulin resistance, diabetes and cognitive function: Consequences for preventative strategies. Diabetes Metab..

[71] Espeland M.A., Lipska K., Miller M.E., Rushing J., Cohen R.A., Verghese J., McDermott M.M., King A.C., Strotmeyer E.S., Blair S.N. (2017). Effects of Physical Activity Intervention on Physical and Cognitive Function in Sedentary Adults With and Without Diabetes. J. Gerontol. Ser. A.

[72] Eakin K.A., Saleem M., Herrmann N., Cogo-Moreira H., Mielke M.M., Oh P.I., Haughey N.J., Venkata S.L.V., Lanctôt K.L., Swardfager W. (2019). Plasma Sphingolipids Mediate a Relationship between Type 2 Diabetes and Memory Outcomes in Patients with Coronary Artery Disease Undertaking Exercise. J. Alzheimer’s Dis..

[73] Shellington E.M., Reichert S.M., Heath M., Gill D.P., Shigematsu R., Petrella R.J. (2018). Results From a Feasibility Study of Square-Stepping Exercise in Older Adults With Type 2 Diabetes and Self-Reported Cognitive Complaints to Improve Global Cognitive Functioning. Can. J. Diabetes.

[74] Olson E.A., Mullen S.P., Raine L.B., Kramer A.F., Hillman C.H., McAuley E. (2017). Integrated social- and neuro-cognitive model of physical activity behavior in older aduts with metabolic disease. Physiol. Behav..

[75] Lin H., Yuan Y., Tian S., Han J., Huang R., Guo D., Wang J., An K., Wang S. (2019). In Addition to Poor Glycemic Control, a High Level of Irisin in the Plasma Portends Early Cognitive Deficits Clinically in Chinese Patients With Type 2 Diabetes Mellitus. Front. Endocrinol..

[76] Wang K., Song F., Xu K., Liu Z., Han S., Li F., Sun Y. (2019). Irisin attenuates neuroinflammation and prevents the memory and cognitive deterioration in streptozotocin-induced diabetic mice. Mediat. Inflamm..

[77] Wang S., Pan J. (2016). Irisin ameliorates depressive-like behaviors in rats by regulating energy metabolism. Biochem. Biophys. Res. Commun..

[78] Han W., Zhang C., Wang H., Yang M., Guo Y., Li G., Zhang H., Wang C., Chen D., Geng C. (2019). Alterations of irisin, adropin, preptin and BDNF concentrations in coronary heart disease patients comorbid with depression. Ann. Transl. Med..

[79] Hou Z., Zhang J., Yu K., Song F. (2020). Irisin ameliorates the postoperative depressive-like behavior by reducing the surface expression of epidermal growth factor receptor in mice. Neurochem. Int..

[80] Knapen J., Vancampfort D., Moriën Y., Marchal Y. (2015). Exercise therapy improves both mental and physical health in patients with major depression. Disabil. Rehabil..

[81] Liu W., Zhai X., Li H., Ji L. (2014). Depression-like behaviors in mice subjected to co-treatment of high-fat diet and corticosterone are ameliorated by AICAR and exercise. J. Affect. Disord..

[82] Uysal N., Yuksel O., Kizildag S., Yuce Z., Gumus H., Karakilic A., Guvendi G., Koc B., Kandis S., Ates M. (2018). Regular aerobic exercise correlates with reduced anxiety and incresed levels of irisin in brain and white adipose tissue. Neurosci. Lett..

[83] Siddiqui F., Lindblad U., Bennet L. (2014). Physical inactivity is strongly associated with anxiety and depression in Iraqi immigrants to Sweden: A cross-sectional study. BMC Public Health.

[84] Dinel A.-L., André C., Aubert A., Ferreira G., Layé S., Castanon N. (2011). Cognitive and emotional alterations are related to hippocampal inflammation in a mouse model of metabolic syndrome. PLoS ONE.

[85] Di Liegro C.M., Schiera G., Proia P., Liegro I. (2020). Di Physical activity and brain health. Genes.

[86] Cassilhas R.C., Tufik S., De Mello M.T. (2016). Physical exercise, neuroplasticity, spatial learning and memory. Cell. Mol. Life Sci..

[87] Garber C.E., Blissmer B., Deschenes M.R., Franklin B.A., Lamonte M.J., Lee I.M., Nieman D.C., Swain D.P. (2011). Quantity and quality of exercise for developing and maintaining cardiorespiratory, musculoskeletal, and neuromotor fitness in apparently healthy adults: Guidance for prescribing exercise. Med. Sci. Sports Exerc..

[88] Pedersen B.K., Saltin B. (2015). Exercise as medicine—Evidence for prescribing exercise as therapy in 26 different chronic diseases. Scand. J. Med. Sci. Sport..

[89] Qaisar R., Bhaskaran S., Van Remmen H. (2016). Muscle fiber type diversification during exercise and regeneration. Free Radic. Biol. Med..

[90] World Health Assembly Resolution WHA57.17 (2004). Physical Activity and Older Adults.

[91] Haskell W.L., Lee I.-M., Pate R.R., Powell K.E., Blair S.N., Franklin B.A., Macera C.A., Heath G.W., Thompson P.D., Bauman A. (2007). Physical activity and public health: Updated recommendation for adults from the American College of Sports Medicine and the American Heart Association. Circulation.

[92] Care D. (2020). ADA Standards of medical care in diabetes—2020. Diabetes Care.

[93] Franz M.J., Boucher J.L., Rutten-Ramos S., VanWormer J.J. (2015). Lifestyle Weight-Loss Intervention Outcomes in Overweight and Obese Adults with Type 2 Diabetes: A Systematic Review and Meta-Analysis of Randomized Clinical Trials. J. Acad. Nutr. Diet..

[94] Baigent C., Keech A., Kearney P.M., Blackwell L., Buck G., Pollicino C., Kirby A., Sourjina T., Peto R., Collins R. (2005). Efficacy and safety of cholesterol-lowering treatment: Prospective meta-analysis of data from 90 056 participants in 14 randomised trials of statins. Lancet.

[95] Lee S.S., Yoo J.H., Kang S.H., Woo J.H., Shin K.O., Kim K.B., Cho S.Y., Roh H.T., Il Kim Y. (2014). The Effects of 12 Weeks Regular Aerobic Exercise on Brain-derived Neurotrophic Factor and Inflammatory Factors in Juvenile Obesity and Type 2 Diabetes Mellitus. J. Physiol. Ther. Sci..

[96] Da Marques N.S.F., de Abreu L.C., dos Santos B.V., Neto C.F.R., da Silva J.R.C., de Braga K.K.S., da Uchôa K.S., Moraes L.M.S., de Ferreira L.C.P., Ribeiro N.G. (2018). Cardiorespiratory parameters and glycated hemoglobin of patients with type 2 diabetes after a rehabilitation program. Medicine.

[97] Di Battista A.P., Moes K.A., Shiu M.Y., Hutchison M.G., Churchill N., Thomas S.G., Rhind S.G. (2018). High-Intensity Interval Training Is Associated With Alterations in Blood Biomarkers Related to Brain Injury. Front. Physiol..

[98] Freitas D., Rocha-vieira E., de Sousa R.A.L., Alvarenga B., Rocha-gomes A., Garcia B., Cassilhas R.C., Mendonça V.A., Camargos A.C.R., Lacerda A.C. (2019). High-intensity interval training improves cerebellar antioxidant capacity without affecting cognitive functions in rats. Behav. Brain Res..

[99] Gibala M.J., McGee S.L. (2008). Metabolic Adaptations to Short-term High-Intensity Interval Training: A Little Pain For a Lot of Gain?. Exerc. Sport Sci. Rev..

[100] Little J.P., Safdar A., Wilkin G.P., Tarnopolsky M.A., Gibala M.J. (2010). A practical model of low-volume high-intensity interval training induces mitochondrial biogenesis in human skeletal muscle: Potential mechanisms. J. Physiol..

[101] Mendes R., Sousa N., Themudo-Barata J.L., Reis V.M. (2019). High-intensity interval training versus moderate-intensity continuous training in middle-aged and older patients with type 2 diabetes: A randomized controlled crossover trial of the acute effects of treadmill walking on glycemic control. Int. J. Environ. Res. Public Health.

[102] Roden M. (2012). Exercise in type 2 diabetes: To resist or to endure?. Diabetologia.

[103] VanDijk J., Manders R., Tummers K., Bonomi A., Stehouwer C., Hartgens F., VanLoon L. (2012). Both resistance- and endurance-type exercise reduce the prevalence of hyperglycaemia in individuals with impaired glucose tolerance and in insulin-treated and non-insulin-treated type 2 diabetic patients. Diabetologia.

[104] Afzalpour M.E., Chadorneshin H.T., Foadoddini M., Eivari H.A. (2015). Comparing interval and continuous exercise training regimens on neurotrophic factors in rat brain. Physiol. Behav..

[105] Jamurtas A.Z., Fatouros I.G., Deli C.K., Georgakouli K., Poulios A., Draganidis D., Papanikolaou K., Tsimeas P., Chatzinikolaou A., Avloniti A. (2018). The effects of acute low-volume HIIT and aerobic exercise on leukocyte count and redox status. J. Sport. Sci. Med..

[106] Holloway T.M., Bloemberg D., Da Silva M.L., Simpson J.A., Quadrilatero J., Spriet L.L. (2015). High intensity interval and endurance training have opposing effects on markers of heart failure and cardiac remodeling in hypertensive rats. PLoS ONE.

